# Effects on Adherence to a Mobile App–Based Self-management Digital Therapeutics Among Patients With Coronary Heart Disease: Pilot Randomized Controlled Trial

**DOI:** 10.2196/32251

**Published:** 2022-02-15

**Authors:** Yuxi Li, Yanjun Gong, Bo Zheng, Fangfang Fan, Tieci Yi, Yimei Zheng, Pengkang He, Jin Fang, Jia Jia, Qin Zhu, Jie Jiang, Yong Huo

**Affiliations:** 1 Department of Cardiology Peking University First Hospital Beijing China; 2 Institute of Cardiovascular Disease Peking University First Hospital Beijing China; 3 Hypertension Precision Diagnosis and Treatment Research Center Peking University First Hospital Beijing China; 4 Stragety & New Business Development of Philips Greater China Shanghai China

**Keywords:** coronary heart disease, secondary prevention, self-management, mobile app, adherence, digital therapeutics, mobile phone

## Abstract

**Background:**

The adherence to secondary prevention treatment in patients with coronary heart disease (CHD) is low. Digital therapeutics (DTx) refers to an emerging branch of medicine that delivers medical interventions directly to patients using evidence-based, clinically evaluated, technology-based software algorithms or apps to facilitate disease management, which may be an efficient tool to optimize adherence.

**Objective:**

This paper aims to investigate the effect of mobile app–based self-management DTx on long-term use of secondary prevention medications in patients with CHD in China.

**Methods:**

This pilot study was a parallel-designed, open-labeled, single-center, randomized controlled trial. Hospitalized patients with CHD admitted to Peking University First Hospital between April 2016 and June 2017 were randomized before discharge on a 1:1 ratio. The intervention group received regular follow-up combined with DTx, which is a self-management mobile app already installed on an Android 5 (Mi Pad 1, Xiaomi Corporation) tablet. Structured data from the hospital informatics system were integrated automatically, and medication, lifestyle intervention plan, follow-up protocol, and patient education materials were also provided according to the diagnosis. Participants could use DTx for self-management at home. The control group was under conventional hospital–based follow-up care. Patients were followed up for 1 year, and the primary end point was the percentage of all guideline-recommended medications at 12 months. The secondary end points included the percentage adhered to standard secondary prevention medications at 6 months, the control rate of lipid profile, and blood pressure at 6 months and 1 year.

**Results:**

Among 300 randomized patients with CHD, 290 (96.7%) were included in the final analysis, including 49.3% (143/290) and 50.7% (147/290) of patients from the intervention and control groups, respectively. Baseline characteristics were similar between the 2 groups. There was a statistically significant improvement in the percentage of all guideline-recommended medications at 12 months in the intervention group compared with the control group (relative risk [RR] 1.34, 95% CI 1.12-1.61; *P*=.001), and there was no interaction with baseline characteristics. The intervention group had a significantly higher proportion of patients achieving blood pressure under control (systolic blood pressure <140 mm Hg and diastolic blood pressure <90 mm Hg) and low-density lipoprotein cholesterol <1.8 mmol/L (RR 1.45, 95% CI 1.22-1.72; *P*<.001 and RR 1.40, 95% CI 1.11-1.75; *P*=.004, respectively) at 12 months. Furthermore, on logistic regression, the intervention group had a lower risk of withdrawing from guideline-recommended medications (odds ratio 0.46, 95% CI 0.27-0.78; *P*=.004).

**Conclusions:**

Among patients with CHD, using a mobile app–based self-management DTx in addition to traditional care resulted in a significant improvement in guideline-recommended medication adherence at 12 months. The results of the trial will be applicable to primary care centers, especially in rural areas with less medical resources.

**Trial Registration:**

ClinicalTrials.gov NCT03565978; https://clinicaltrials.gov/ct2/show/NCT03565978

## Introduction

Coronary heart disease (CHD) is one of the leading causes of death globally, especially in China; the trend of mortality due to CHD is still increasing and the recurrence rate of major cardiovascular events remains high [1]. The long-term use of secondary prevention medications is widely recommended by the national and international guidelines, and proven to improve the prognosis [2-4].

However, the adherence rate to long-term secondary prevention therapies only varies from 30% to 50%, and it is even worse in limited-income countries [5-8], which shows the big gap between the real-world practice and the recommended guidelines and, thus, being a key challenge limiting the overall benefits of these therapies. Poor adherence has been demonstrated to be associated with a 50% to 80% relative increase in mortality and increased health care cost [9,10].

The causes of nonadherence are complex and not due to a single reason only; however, a good medical system with primary care support and a long-term follow-up plan, including cardiac rehabilitation programs, for patients with CHD, especially those discharged from a hospital, is crucial. For overpopulated countries, such as China, the lack of specialized cardiac rehabilitation staff is one of the main reasons for poor adherence [5,11]. A novel and effective management model other than conventional, hospital-based follow-up interventions is needed to optimize the long-term treatment of CHD. Recent advances in digital therapeutics (DTx), which delivers medical interventions directly to patients using evidence-based, clinically evaluated, technology-based software algorithms or apps to facilitate disease management, such as smartphones and technology, have made DTx a promising solution for secondary prevention management of chronic diseases [12-14]. SMS text messaging is one of the mobile health (mHealth) approaches that have been demonstrated to significantly reduce the low-density lipoprotein cholesterol (LDL-C) level, systolic blood pressure (SBP), and BMI of patients with CHD [15,16]. Recently published systematic review articles have shown that mHealth significantly improved patients’ cardiovascular risk factors rather than mortality for secondary prevention; however, among the trials included in the meta-analysis, only a very few studies were conducted in limited-income countries [17,18].

Furthermore, patient self-management is one of the key issues in the long-term management of secondary prevention among patients with CHD [7]. This study aims to explore, in a randomized controlled trial, the effect of a mobile app designed for self-management DTx at home on the long-term use of secondary prevention medications in patients with CHD in China.

## Methods

### Study Design

This BAMA (name of a famous *longevity village* in the northwest of Guangxi province, China) pilot study was a parallel-designed, open-labeled, single-center, randomized controlled trial conducted in Peking University First Hospital, China. Patients with CHD were randomized to receive conventional hospital-based care and management (control group) or in addition to use a mobile app for self-management DTx (intervention group). Patients were followed up for 1 year. The objective measures of cardiovascular risk factors, including LDL-C level, blood pressure, and the percentage of patients who were taking guideline-recommended medications at 6 months and 1 year postrandomization were compared between the 2 groups.

Patients provided written informed consent, and the study protocol, in compliance with the principles outlined in the Declaration of Helsinki, was approved by the Institutional Ethics Committee of Peking University First Hospital and registered at ClinicalTrials.gov (NCT03565978).

### Study Population

The patients were enrolled into the study only if they met all of the following criteria: (1) were male or female aged ≥18 years; (2) were diagnosed with CHD (defined as documented prior myocardial infarction, coronary artery bypass graft surgery, percutaneous coronary intervention, or ≥50% stenosis in at least one major epicardial vessel on coronary angiography); (3) were willing to undergo a self-management intervention and comply with the follow-up plan; (4) had basic reading skills in Chinese; and (5) voluntarily participated in the study and signed the informed consent form. Patients were excluded if they met one of the following criteria: (1) already enrolled in another interventional clinical trial, (2) refused to sign the informed consent or withdrew from the study for any specific reasons, (3) had cognitive disorder and were unable to communicate normally, and (4) had limited basic mobile technology skills to operate a mobile app after training.

**Figure 1:**
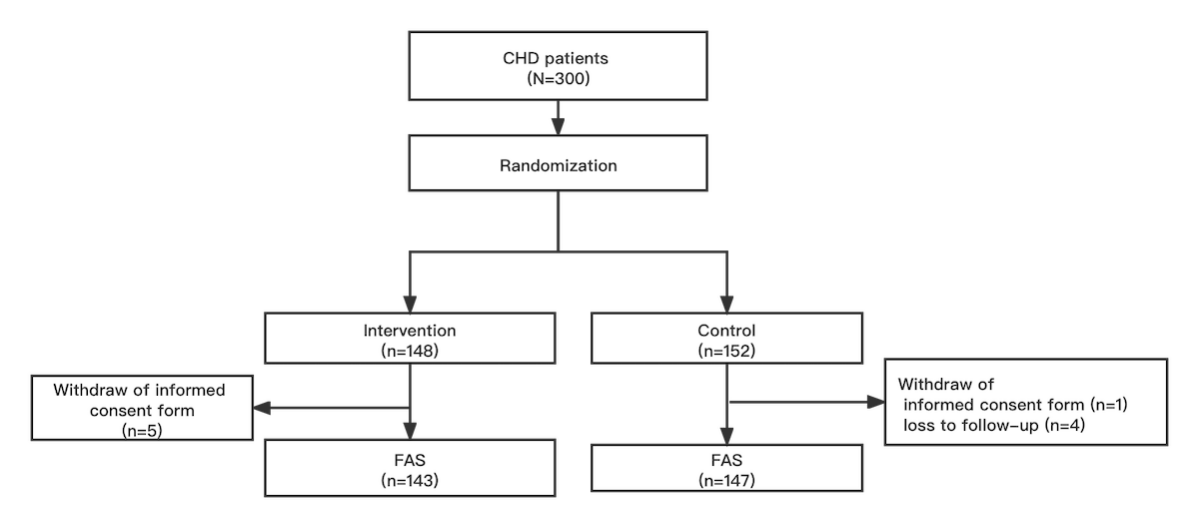
Flowchart of the study participants. CHD: coronary heart disease; FAS: full analysis set.

Recruitment was performed between April 2016 and June 2017, and the follow-up continued until June 2018. The participant flowchart is shown in Figure 1. All participants were recruited before discharge from the hospital, after being hospitalized because of CHD. A comparison between recruited participants and hospitalized patients with CHD during the same period who did not participate in this trial can be found in Table S1 in Multimedia Appendix 1. Enrollment of participants was continued until the planned sample size was reached.

### Group Allocation and Intervention

Randomization was conducted using a computerized web-based randomization program. The random allocation sequence was in a uniform 1:1 allocation ratio. Patients were randomized to the intervention group, which comprised participants who underwent regular follow-up combined with a mobile app–based self-management DTx, or the control group, which comprised participants who only received regular follow-up care and patient education. The control group was treated and followed up as usual. Printed patient education materials were given to patients at the beginning of the study and during each visit, and the same content was sent to the intervention group through the self-management mobile app.

The DTx for the intervention group is a self-management mobile app already installed on an Android tablet (provided by the study), and the app could also be installed on iOS or smartphones. The DTx system includes a physician portal, a health manager portal, and a patient portal and contains 3 modules. The first one is the discharge module, which was used before discharge. Structured data from the hospital informatics system were integrated automatically in the DTx system, and trial staff confirmed the medication and lifestyle intervention plan and arranged follow-up protocol in the DTx system and educated participants on how to use the patient portal. Patient education materials and instructions on medication were also provided in the app according to the diagnosis at discharge.

The second module is the home management module. An electronic blood pressure meter was given to all participants at the beginning of the study to encourage self-management. The intervention group had their blood pressure and heart rate data transferred through Bluetooth connection to the app, or they could also input the data on the app by themselves. In addition, an automatic alarm was set up in the patient portal to help manage their daily medical regiment. Participants in the intervention group could also record symptoms and notes in the DTx app, whereas the control group was asked to record their data manually on the patients’ education book. All data recorded by the home management module are shared with trial staff physicians and nurses in the physician portal.

The third module is the follow-up module, which was used during the follow-up; the DTx had a dynamic design and dashboard overview displaying the latest discharge summaries, vital signs, symptoms, and medications. Physicians could update medication and lifestyle plans during each follow-up in the DTx, whereas participants in the control group used traditional electronic health records. An illustrated description slide is provided in Multimedia Appendix 2.

### Trial Procedures

In all participants, baseline demographic characteristics, experience in smartphone use, access to Wi-Fi at home, prior medical history, and tobacco use were assessed by nurses during their hospitalization. Blood pressure, heart rate, and BMI were measured according to standardized procedures. Blood pressure and heart rate were measured 3 times using an automatic Omron device (Omron Healthcare, Inc), and mean readings were used for the analyses. Plasma creatinine, LDL-C, and hemoglobin A_1c_ levels were analyzed at local laboratories. The estimated glomerular filtration rate was calculated based on the Modification of Diet in Renal Disease equation [19]. The left ventricular ejection fraction was measured by certified cardiologists. The Teich method was used, unless the measurement based on the Simpson method was used if the patient had prior myocardial infarction or significant ventricle dilation.

An invasive diagnostic coronary angiography was performed during the hospitalization, and the secondary prevention treatment regimen was according to the discretion of the physician based on the patients’ specific clinical condition. All patients were asked to visit the outpatient clinic every 3 months, which was also the routine follow-up plan for patients with coronary diseases in this hospital. Blood pressure and heart rate were measured during each follow-up visit according to the aforementioned standard procedure. LDL-C levels were measured at the 3-, 6-, and 12-month follow-up visits at the local laboratories.

The primary end point of the study was the percentage of patients who used the standard guideline-recommended secondary prevention medications at 1 year after enrollment. This medication regimen included (1) aspirin, P2Y12 receptor inhibitor, or both in patients who had acute coronary syndrome or percutaneous coronary intervention during hospitalization; (2) statins; and (3) angiotensin-converting enzyme inhibitors or angiotensin-receptor blockers and β-receptor blockers for patients with a history of myocardial infarction or heart failure; this regimen was provided to the patients if there was not any contraindication. During the follow-up visit at 6 and 12 months, the physicians obtained information on whether the patients were using each of the medications.

The secondary efficacy end points of the study included the percentage of patients who took standard secondary prevention medications at 6 months and the control rate of lipid profile (LDL-C <1.8 mmol/L) and blood pressure (SBP <140 mm Hg and diastolic blood pressure [DBP] <90 mm Hg) at 6 months and 1 year after enrollment.

### Statistical Analysis

According to previous data, the percentage of patients who used the standard guideline-recommended secondary prevention medications was approximately 60% when there was no intervention. The proportion in the intervention group was expected to increase to 75%; thus, 152 participants were needed for each group to have a power of 80% (2-tailed and at a 5% significance level) to detect the difference. All intervention evaluations were performed on the principle of per-protocol analysis. Participants were analyzed by the original assigned groups.

Description of continuous variables are summarized as means and SDs unless skewed and then presented as medians and IQRs. Categorical variables are presented as frequencies and percentages.

For the primary and categorical secondary end points, the proportion of patients with a positive primary end point was compared between the 2 randomized groups (intervention and control) using a log-binomial regression, and the result was presented as relative risk (RR) and 95% CI. Similarly, the mean differences in blood pressure, heart rate, and LDL-C levels were compared between the 2 randomized groups (intervention and control) using a log-binomial regression, and the result was presented as the mean difference and 95% CI. A logistic regression analysis was made to compare the rate of withdrawal from the guideline-recommended medications within the 12-month follow-up period between the 2 groups, and the result was presented as odds ratio and 95% CI. The analyses were otherwise unadjusted. Subgroup analyses were conducted by sex, age, BMI, current tobacco use, prior history of hypertension, dyslipidemia, left ventricular ejection fraction, and discharge diagnoses of stable CHD versus acute coronary syndrome using logistic regression.

Data were collected during each follow-up visit. Two individuals with experience in data entry independently entered all data into separate EpiData (version 3.1, EpiData Software) databases. The 2 databases were compared, and discrepancies were resolved by checking the original data. Data quality monitoring was conducted by an independent monitoring staff. If necessary, data queries were also made.

Analyses were conducted using R (version 3.6.2; R Foundation for Statistical Computing) [20] and RStudio (version 1.2.5033; RStudio, Inc) [21]. All statistical tests were 2-tailed, and a 5% significance threshold was maintained.

## Results

### Overview

Between April 8, 2016, and June 28, 2017, 300 patients who met the inclusion and exclusion criteria were enrolled in the study. Figure 1 illustrates the flow diagram of the participant recruitment, randomization, and waning throughout the trial. As mentioned above, given that the recruitment was based on the willingness of patients and their ability to handle smartphones, it was difficult to determine the number of potential patients who met the inclusion and exclusion criteria. A comparison between recruited patients and hospitalized patients with CHD during the same period who did not participate in this trial is shown in Table S1 in Multimedia Appendix 1, and the results showed no significant differences in the baseline characteristics between the 2 groups. Randomization yielded 49.3% (148/300) and 50.7% (152/300) patients in the intervention and control groups, respectively. However, 2% (6/300) of the patients withdrew consent after randomization, and 1.3% (4/300) of the patients were lost to follow-up during the study. Of the 300 patients, 290 (96.7%) patients were followed up for 12 months after randomization.

As shown in Table 1, the mean age 61.38 (SD 8.88) years versus 62.27 (SD 9.87) years (*P*=.42) and sex proportion (29/143, 20.3% vs 36/147, 24.5%, female sex; *P*=.47) between the 2 groups were similar. Other baseline characteristics, including prior medical history, cardiac risk factors, laboratory tests, CHD presentation, and angiographic features, were all similar between the 2 groups.

**Table 1:** Patients’ baseline characteristics.

		All (n=290)	Intervention (n=143)	Control (n=147)	*P* value	
**Demographics**						
	Female sex, n (%)	65 (22.4)	29 (20.3)	36 (24.5)	.47	
	Age (years), mean (SD)	61.83 (9.39)	61.38 (8.88)	62.27 (9.87)	.42	
	Han, n (%)	271 (94.1)	137 (96.5)	134 (91.8)	.11	
	Never used a smartphone, n (%)	25 (8.8)	7 (5)	18 (12.4)	.15	
	Wi-Fi available at home, n (%)	264 (92.6)	131 (93.6)	133 (91.7)	.71	
**Prior medical history, n (%)**						
	Myocardial infarction	48 (16.6)	21 (14.7)	27 (18.4)	.49	
	Heart failure	12 (4.1)	8 (5.6)	4 (2.7)	.35	
	Prior PCI^a^	80 (27.6)	40 (28)	40 (27.2)	.99	
	Prior CABG^b^	4 (1.4)	3 (2.1)	1 (0.7)	.60	
	Hypertension	196 (67.6)	99 (69.2)	97 (66)	.64	
	Diabetes	131 (45.2)	68 (47.6)	63 (42.9)	.49	
	Dyslipidemia	176 (60.7)	86 (60.1)	90 (61.2)	.95	
	Renal insufficiency	26 (9)	11 (7.7)	15 (10.2)	.59	
	Current dialysis	4 (1.4)	3 (2.1)	1 (0.7)	.60	
	Cerebral disease	35 (12.1)	13 (9.1)	22 (15)	.18	
	Peripheral artery disease	5 (1.7)	1 (0.7)	4 (2.7)	.38	
	Current tobacco use (<1 year)	179 (61.7)	89 (62.2)	90 (61.2)	.61	
	Premature CHD^c^ family history	36 (12.4)	19 (13.3)	17 (11.6)	.25	
**Physical examination**						
	BMI (kg/m^2^), mean (SD)	26.11 (3.27)	25.93 (3.07)	26.28 (3.46)	.37	
	BMI>24 (kg/m^2^), n (%)	218 (76)	109 (77.9)	109 (74.2)	.55	
	SBP^d^ (mm Hg), mean (SD)	135.70 (20.03)	134.43 (18.48)	136.93 (21.42)	.29	
	DBP^e^ (mm Hg), mean (SD)	78.31 (12.43)	77.74 (11.35)	78.86 (13.42)	.44	
	HR^f^ (bpm^g^), mean (SD)	70.84 (10.07)	70.09 (9.39)	71.58 (10.67)	.21	
**Laboratory results**						
	eGFR^h^ (mL/min × 1.73 m²), mean (SD)	76.77 (19.27)	76.33 (18.09)	77.19 (20.40)	.70	
	eGFR≥60 (mL/min × 1.73 m²), n (%)	245 (84.5)	122 (85.3)	123 (83.7)	.82	
	LDL^i^ (mmol/L), mean (SD)	2.47 (0.86)	2.41 (0.88)	2.53 (0.85)	.25	
	HbA_1c_^j^ (%), mean (SD)	6.89 (1.55)	6.96 (1.58)	6.82 (1.52)	.48	
	LVEF^k^ (%), mean (SD)	67.01 (10.48)	66.91 (11.01)	67.12 (9.97)	.87	
**Discharge diagnoses, n (%)**						.80
	Stable angina	54 (18.6)	25 (17.5)	29 (19.7)		
	Unstable angina	181 (62.4)	92 (64.3)	89 (60.5)		
	Acute myocardial infarction	55 (19)	26 (18.2)	29 (19.7)		
**Coronary artery lesions, n (%)**						.20
	Single-vessel lesions	100 (34.5)	55 (38.5)	45 (30.6)		
	Double-vessel lesions	90 (31)	43 (30.1)	47 (32)		
	Triple-vessel lesions	98 (33.8)	43 (30.1)	55 (37.4)		
	Left main lesion	2 (0.7)	2 (1.4)	0 (0)		
Number of stents, median (quartile 1, quartile 3)		2 (1, 2)	2 (1, 2)	2 (1, 2.5)	.50	
Full revascularization, n (%)		192 (66.2)	96 (67.1)	96 (65.3)	.84	

^a^PCI: percutaneous coronary intervention.

^b^CABG: coronary artery bypass grafting.

^c^CHD: coronary heart disease.

^d^SBP: systolic blood pressure.

^e^DBP: diastolic blood pressure.

^f^HR: heart rate.

^g^bpm: beat per minute.

^h^eGFR: estimated glomerular filtration rate.

^i^LDL: low-density lipoprotein.

^j^HbA_1c_: hemoglobin A_1c_.

^k^LVEF: left ventricular ejection fraction.

### Primary Outcome

As shown in Table 2, there was a statistically significant improvement in the percentage of patients using all guideline-recommended medications at 12 months in the intervention group compared with the control group (RR 1.34, 95% CI 1.12-1.61; *P*=.001). As shown in Table S2 in Multimedia Appendix 1, similar results were found for aspirin and P2Y12 receptor inhibitors. Regarding statin use, there was a marginal improvement observed in the intervention group compared with the control group; however, there were no differences in angiotensin-converting enzyme inhibitor or angiotensin-receptor blocker or β-receptor blocker use between the 2 groups. However, there was no interaction between baseline characteristics and intervention for the primary outcome in all subgroups (Multimedia Appendix 1, Figure S1).

The intervention group had a significantly higher proportion of patients with controlled blood pressure (SBP <140 mm Hg and DBP <90 mm Hg) and LDL-C level <1.8 mmol/L (RR 1.45, 95% CI 1.22-1.72; *P*<.001 and RR 1.40, 95% CI 1.11-1.75; *P*=.004, respectively) at 12 months compared with the control group. At 6 months, significant differences in blood pressure levels (RR 1.38, 95% CI 1.16-1.64; *P*<.001) were found between the 2 groups; however, no significant difference in LDL-C levels was found (RR 1.18, 95% CI 0.96-1.46; *P*=.12; Table 2).

**Table 2:** Secondary prevention medication and risk factor control between the 2 groups (N=290).

			Intervention (n=143)		Control (n=147)		Relative risk (95% CI)		*P* value
**Primary end point**									
	All medications at 12 months, n (%)	103 (72)		79 (53.7)		1.34 (1.12-1.61)		.001	
	Adherence score at 12 months, mean (SD)	0.72 (1.18)		0.69 (1.18)		0.71 (0.77-1.25)		.81	
**Secondary end point, n (%)**									
	All medications at 6 months	121 (86.4)		101 (69.2)		1.25 (1.10-1.42)		<.001	
	Adherence score at 6 months	1.08 (1.45)		1.01 (1.26)		1.05 (0.58-1.36)		.69	
**Risk factors at 12 months, n (%)**									
	LDL^a^<1.8 mmol/L	87 (60.8)		64 (43.5)		1.40 (1.11-1.75)		.004	
	SBP^b^<140 mm Hg and DBP^c^<90 mm Hg	99 (82.5)		75 (56.8)		1.45 (1.22-1.72)		<.001	
**Risk factors at 6 months, n (%)**									
	LDL<1.8 mmol/L	85 (59.4)		74 (50.3)		1.18 (0.96-1.46)		.12	
	SBP<140 mm Hg and DBP<90 mm Hg	96 (67.1)		77 (52.4)		1.38 (1.16-1.64)		<.001	

^a^LDL: low-density lipoprotein.

^b^SBP: systolic blood pressure.

^c^DBP: diastolic blood pressure.

### Change in Primary End Point From Baseline to the End of 12 Months

**Figure 2:**
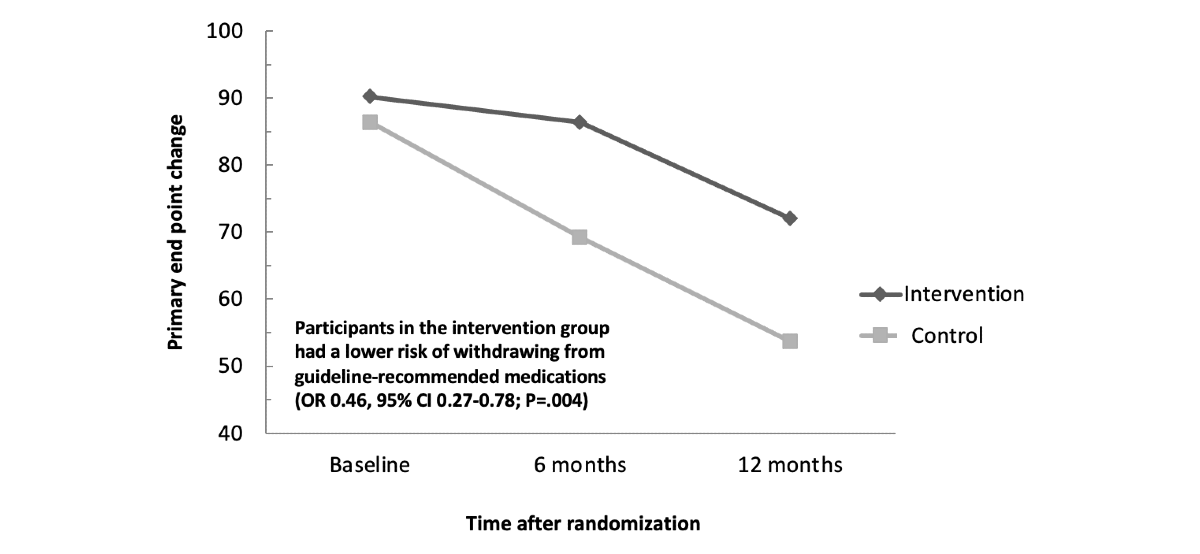
Proportion of patients taking the standard secondary prevention medications. OR: odds ratio.

Figure 2 shows the changing trends of primary outcome measures at baseline and at 6 and 12 months between the 2 groups. Participants in both the intervention and control groups had poor adherence to key secondary prevention medications during the 1-year follow-up duration; however, the proportion of patients on standard medications was higher in the intervention group than in the control group. On logistic regression, participants in the intervention group had a lower risk of withdrawal from the guideline-recommended medications (odds ratio 0.46, 95% CI 0.27-0.78; *P*=.004).

**Figure 3:**
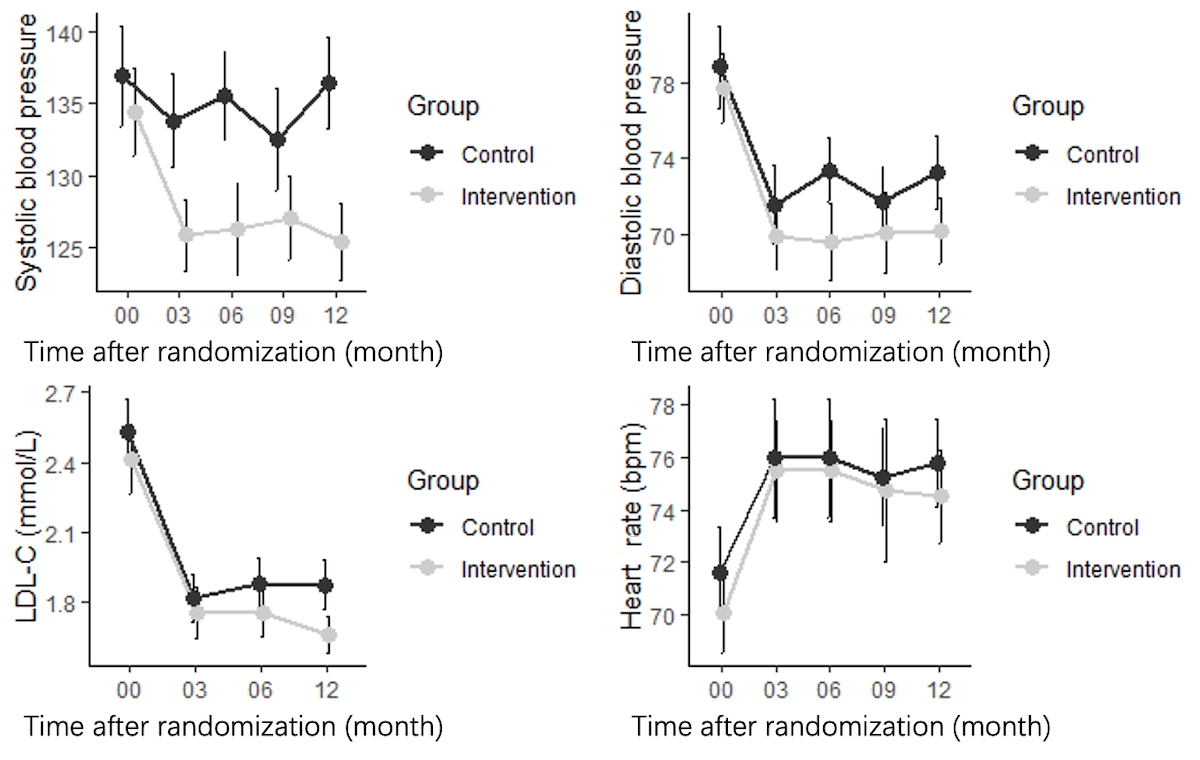
Blood pressure and low-density lipoprotein cholesterol (LDL-C) level changes between the 2 groups. bpm: beats per minute.

As shown in Figure 3, the trends of changes in blood pressure, LDL-C levels, and heart rate were analyzed. During the study, SBP and DBP decreased during the first 3 months in both groups, but the decrease was slightly higher in the intervention group. However, with the decrease in medication adherence, the blood pressure in the control group began to increase, especially for SBP, which almost returned to the baseline values at 12 months. Meanwhile, both SBP and DBP in the intervention group remained well controlled. The same pattern was found in the patients’ lipid profiles. The trend in the changes in heart rate between the 2 groups was similar.

Accordingly, in the linear regression analysis (Table 3), the changes in the values from baseline to the 12-month follow-up for SBP, DBP, and LDL-C levels were all significantly higher in the intervention group than in the control group (change in SBP −11 mm Hg, 95% CI −15 to −7; *P*<.001; change in DBP −3 mm Hg, 95% CI −6 to −4; *P*=.02; change in LDL-C level −0.22 mmol/L, 95% CI, −0.34 to −0.09; *P*=.001). However, the change in heart rate was not significantly different between the 2 groups (change in heart rate −1 bpm, 95% CI −4 to −1; *P*=.30).

**Table 3:** The mean differences in blood pressure, heart rate, and low-density lipoprotein cholesterol (LDL-C) level between the 12-month follow-up and baseline values (N=290).

Parameter	Value, mean (95% CI)		Mean difference (95% CI)	*P* value
	Intervention (n=143)	Control (n=147)		
SBP^a^ (mm Hg)	125 (122 to 127)	136 (133 to 139)	−11 (−15 to −7)	<.001
DBP^b^ (mm Hg)	70 (68 to 71)	73 (71 to 75)	−3 (−6 to −4)	.02
HR^c^ (bpm^d^)	74 (72 to 76)	75 (74 to 77)	−1 (−4 to −1)	.30
LDL-C (mmol/L)	1.66 (1.58 to 1.74)	1.87 (1.77 to 1.98)	−0.22 (−0.34 to −0.09)	.001

^a^SBP: systolic blood pressure.

^b^DBP: diastolic blood pressure.

^c^HR: heart rate.

^d^bpm: beat per minute.

## Discussion

### Principal Findings

This study demonstrated that the utility of a mobile app–based self-management DTx intervention for patients with CHD led to a significant increase in adherence to guideline-recommended medications as well as an increase in the proportion of patients with controlled LDL-C and blood pressure during the 12-month follow-up duration. The RR of adherence to all guideline-recommended medications at 12 months between the 2 groups was 34%, which might be mainly attributed to the dual antiplatelet and statin therapy, which is the fundamental secondary prevention treatment for CHD. Furthermore, the effect did not show an interaction with other variables based on the subgroup analysis.

During the trial, the adherence to all guideline-recommended medications showed a decreasing trend with time, regardless of whether an intervention was provided or not. The adherence was lower at the 12-month follow-up than at the 6-month follow-up in both the intervention and control groups; however, the intervention group showed a slower decreasing trend of adherence to guideline-recommended medications at 12 months. The same trend can also be seen in the changes in the values of the risk factors, such as blood pressure and LDL-C, which showed a decreasing trend during the first 3 months in both groups. However, the blood pressure in the control group began to increase thereafter, and SBP almost returned to the baseline values at 12 months, whereas that of the intervention group remained well controlled.

Recently, DTx interventions have arisen as a potential means of modifying health behaviors. A topical study reports the findings of a pivotal trial investigating the efficacy and safety of a self-management DTx (a 12-week intervention followed by a 12-week follow-up) in Japanese patients with untreated essential hypertension (baseline office and ambulatory 24 hours BP ≥140/90 mm Hg and ≥130/80 mm Hg, respectively) [14]. Recent studies have evaluated the effectiveness of SMS text messaging services on the improvement in LDL-C level, blood pressure, BMI, and smoking status among patients with CHD [15]. However, a recent systemic review demonstrated insufficient evidence to draw conclusions on their effectiveness [22], and sufficiently powered, high-quality randomized trials are needed, particularly in developing countries. Although SMS text messaging is simple and less costly, the function is somehow limited, especially for important features, such as patient education, vital signs’ monitoring, and medication or adverse event alert, which are commonly applied to smartphones. Studies on mobile app–based management on secondary prevention for cardiovascular diseases have been reported [18,23], but most of the studies had a short follow-up duration, and only 1 RCT included in a recent meta-analysis had an intervention or follow-up period of 12 months [18]. Given the chronic nature of CHD, the long-term effectiveness and clinical outcomes of these mobile app–based interventions remain to be determined because of the lack of long-term follow-up.

The findings of this pilot study are consistent with those of previous studies. However, our study greatly differed from the previous studies based on the following reasons: our study targeted multiple risk factors rather than individual risk factors; the sample size was larger, and the follow-up duration was longer. Another important difference is that we focused on the trend of the improvements in patients, which resulted from the app of the intervention during the 12-month period. Furthermore, a feasibility study was conducted before the formal trial to ensure that the perceptions and acceptance of mHealth are sufficient in patients with CHD [24]. Given that China is the largest developing country, which is the main battlefield of the future management of noncommunicable diseases including CHD, the positive result of this pilot trial will have more influence on the future health care system and delivery of management for chronic diseases.

The current trial had several limitations. First, this trial was conducted at a single large tertiary hospital in Beijing; thus, it is unclear whether the observed benefits are generalizable. However, a routine traditional follow-up program was already established for discharged patients with CHD at this center, indicating that this mobile app will bring greater benefits in rural areas and more remote communities, where traditional secondary prevention programs are more difficult to access. Second, this study was open labeled because of the difficulty of blind design, and the contact of the nursing staff during recruitment and randomization could influence the adherence and introduce bias. However, the control group was also under a routine traditional face-to-face follow-up program including physicians and nurses, and the frequency of contact between the 2 groups was the same, which could minimize the bias. Third, given that this work is a pilot study, medication adherence, rather than solid outcome, was used as the primary end point, which will be confirmed in future studies with a long-term follow-up duration. Third, further work is needed to evaluate the extent to which this mobile app–based self-management intervention may be useful for patients with CHD to improve their prognosis.

### Conclusions

Among patients with CHD, the use of a mobile app–based self-management intervention in addition to the traditional follow-up care resulted in a significant improvement in the guideline-recommended medication adherence at 12 months, compared with the regular care. The results of the trial will be applicable to primary care centers in China, especially in rural areas with less medical resources. The trial will provide new evidence of the efficacy of an internet-based service model in the secondary prevention management of CHD and may help improve risk factor control.

## Appendix

Multimedia Appendix 1 Sensitivity analysis and subgroup analysis.

Multimedia Appendix 2 Illustrated description slides of the digital therapeutics.

Multimedia Appendix 3 CONSORT-eHEALTH checklist (V 1.6.1).

## Abbreviations

CHD: coronary heart disease
DBP: diastolic blood pressure
DTx: digital therapeutics
LDL-C: low-density lipoprotein cholesterol
mHealth: mobile health
RR: relative risk
SBP: systolic blood pressure
