# Supplementary material for: Effects on Adherence to a Mobile App–Based Self-management Digital Therapeutics Among Patients With Coronary Heart Disease: Pilot Randomized Controlled Trial
Source: JMIR Mhealth Uhealth. 2022 Feb 15;10(2):e32251. doi: 10.2196/32251 (PMC8889473; doi:10.2196/32251)
Supplement: Multimedia Appendix 1 [file mhealth_v10i2e32251_app1.docx]

**Supplementary Table S1. Baseline characteristics between CHD patients enrolled in BAMA trial and those that did not who also underwent PCI procedure during the same time.**

|  | **BAMA at randomization** | **Non-BAMA** | **P value** |
| --- | --- | --- | --- |
|  | **N=300** | **N=1020** |  |
| **Demographics** |  |  |  |
| Female sex, n (%) | 67 (22.56) | 311 (30.49) | 0.01 |
| Age (years), mean (SD) | 59.65 (9.31) | 64.70 (10.73) | <0.001 |
| **Prior medical history** |  |  |  |
| Myocardial infarction, n (%) | 49 (16.50) | 222 (21.76) | 0.058 |
| Heart failure, n (%) | 12 (4.04) | 69 (6.76) | 0.114 |
| Prior PCI, n (%) | 82 (27.61) | 212 (20.78) | 0.016 |
| Prior CABG, n (%) | 4 (1.35) | 67 (6.57) | 0.001 |
| Hypertension, n (%) | 202 (68.01) | 739 (72.45) | 0.156 |
| Diabetes, n (%) | 135 (45.45) | 412 (40.39) | 0.136 |
| Dyslipidemia, n (%) | 180 (60.61) | 631 (61.86) | 0.746 |
| Current tobacco use (<1 year), n (%) | 183 (61.62) | 600 (58.82) | 0.117 |
| **Physical examination** |  |  |  |
| BMI (kg/m^2^), mean (SD) | 26.08 (3.29) | 26.12 (3.32) | 0.862 |
| SBP (mmHg), mean (SD) | 135.86 (20.12) | 134.08 (18.81) | 0.161 |
| DBP (mmHg), mean (SD) | 78.32 (12.34) | 75.94 (11.68) | 0.002 |
| LDL (mmol/L), mean (SD) | 2.47 (0.86) | 2.34 (0.87) | 0.029 |
| LVEF (%), mean (SD) | 67.13 (10.39) | 66.76 (10.63) | 0.602 |
| **Discharge diagnoses, n (%)** |  |  | 0.109 |
| Stable angina | 54 (18.24) | 81 (13.00) |  |
| Unstable angina | 186 (62.84) | 413 (66.29) |  |
| Acute myocardial infarction | 56 (18.92) | 129 (20.71) |  |
| Multiple vessel lesion n, (%) | 196 (65.99) | 659 (64.67) | 0.725 |

**Abbreviations:**

SD, standard deviation; PCI, percutaneous coronary intervention; CABG, coronary artery bypass grafting; CHD, coronary heart disease; BMI, body mass index; SBP, systolic blood pressure; DBP, diastolic blood pressure; HR, heart rate; bpm, beat per minute; eGFR, estimated glomerular filtration rate; LDL, low-density lipoprotein cholesterol; HbA1c, hemoglobin A1c; LVEF, left ventricular ejection fraction.

**Supplementary Table S2. List of secondary prevention medications and risk factor control between the two groups**

|  | Intervention  N=143 | Control  N=147 | Relative risk (95%CI) | P value |
| --- | --- | --- | --- | --- |
| Medications at 12 months |  |  |  |  |
| Aspirin | 141/143 (98.60) | 131/147 (89.12) | 1.11 (1.04,1.17) | < 0.001 |
| P2Y12 receptor inhibitor | 130/143 (90.91) | 111/147 (75.51) | 1.20 (1.08,1.34) | < 0.001 |
| Statin | 140/143 (97.90) | 136/147 (92.52) | 1.06 (1.01,1.11) | 0.030 |
| ACEI/ARB | 123/143 (86.01) | 121/147 (82.31) | 1.04 (0.95,1.15) | 0.388 |
| β blocker | 132/143 (92.31) | 133/147 (90.48) | 1.02 (0.95,1.09) | 0.578 |
| Medications at 6 months |  |  |  |  |
| Aspirin | 140/140 (100.00) | 141/146 (96.58) | 1.035 | NaN |
| P2Y12 receptor inhibitor | 138/140 (98.57) | 132/146 (90.41) | 1.09 (1.03, 1.15) | 0.003 |
| Statin | 135/140 (96.43) | 134/146 (91.78) | 1.05 (0.99, 1.11) | 0.095 |
| ACEI/ARB | 129/140 (92.14) | 127/146 (86.99) | 1.06 (0.98, 1.15) | 0.154 |
| β blocker | 135/140 (96.43) | 135/146 (92.47) | 1.04 (0.99, 1.10) | 0.143 |

**Abbreviations:**

ACEI, angiotensin-converting enzyme inhibitors; ARB, angiotensin-receptor blockers


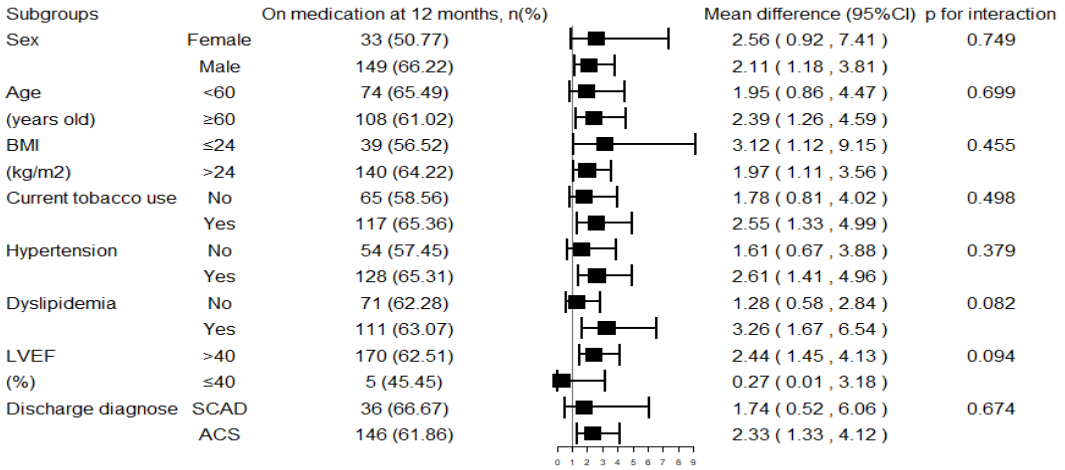


**Supplementary Figure S1. A subgroup and interaction analysis**
