# Supplementary material for: Effects on Adherence to a Mobile App–Based Self-management Digital Therapeutics Among Patients With Coronary Heart Disease: Pilot Randomized Controlled Trial
Source: JMIR Mhealth Uhealth. 2022 Feb 15;10(2):e32251. doi: 10.2196/32251 (PMC8889473; doi:10.2196/32251)
Supplement: Multimedia Appendix 2 [file mhealth_v10i2e32251_app2.pptx]

## Slide 1
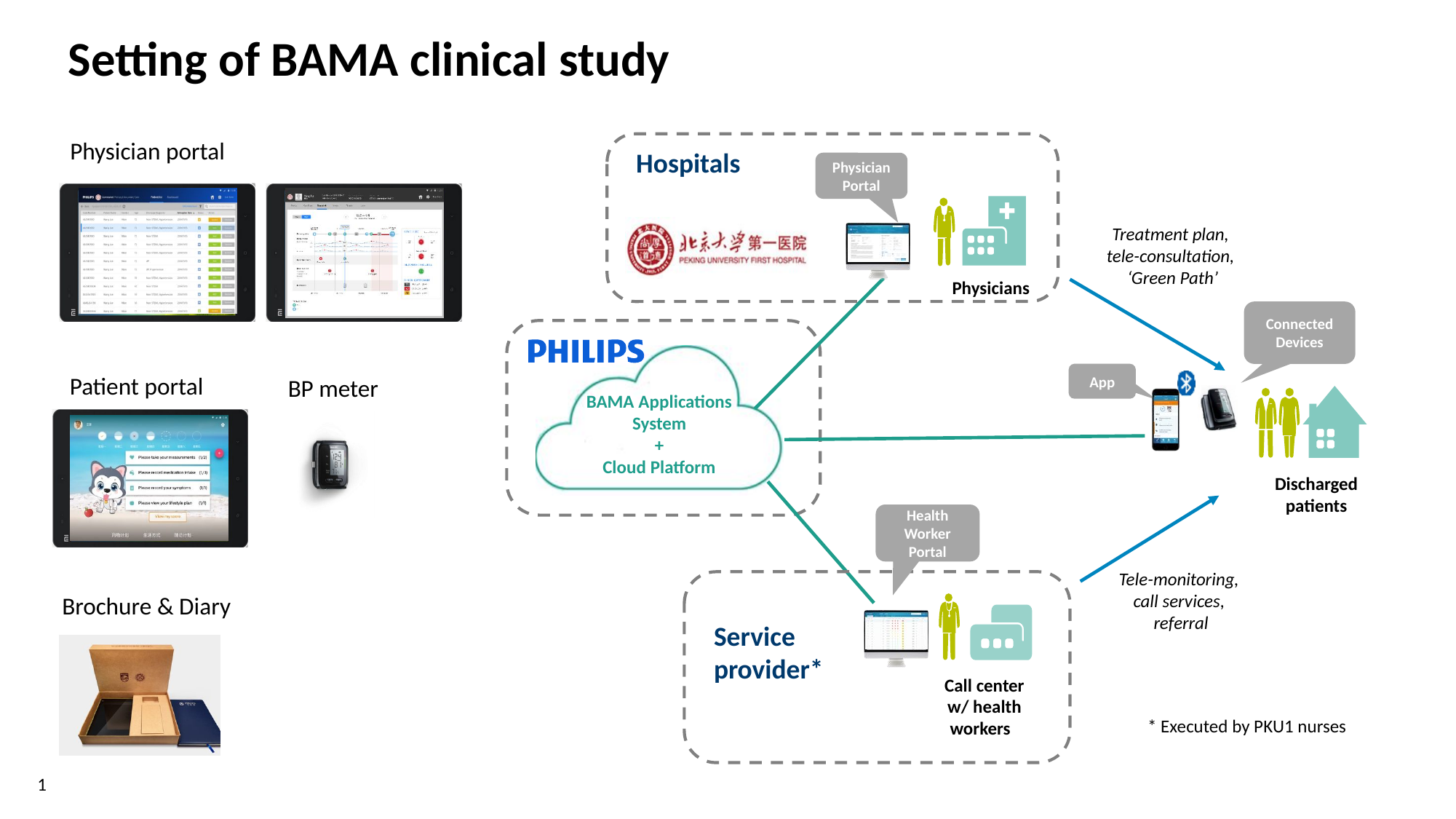

Setting of BAMA clinical study
Physician portal
Hospitals
Physician Portal
Treatment plan,
tele-consultation, ‘Green Path’
Physicians
Connected Devices
App
Patient portal
BP meter
BAMA Applications System
+
Cloud Platform
Discharged patients
Health Worker Portal
Tele-monitoring,
call services,
referral
Brochure & Diary
Service provider*
Call center w/ health workers
* Executed by PKU1 nurses

## Slide 2
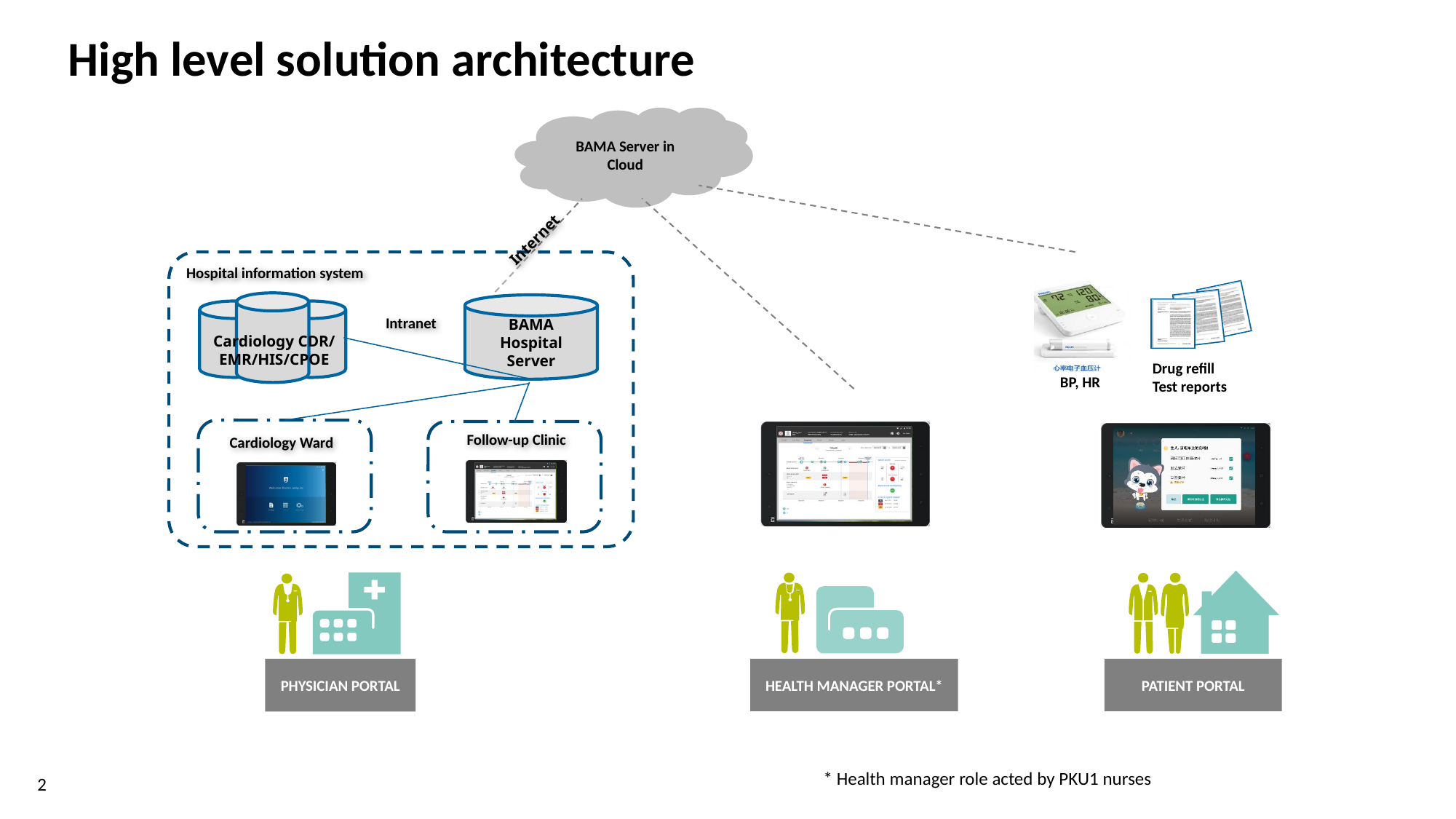

High level solution architecture
BAMA Server in Cloud
Internet
Hospital information system
Cardiology CDR/
EMR/HIS/CPOE
BAMA
Hospital
Server
Intranet
Drug refill
Test reports
BP, HR
Follow-up Clinic
Cardiology Ward
PHYSICIAN PORTAL
HEALTH MANAGER PORTAL*
PATIENT PORTAL
* Health manager role acted by PKU1 nurses

## Slide 3
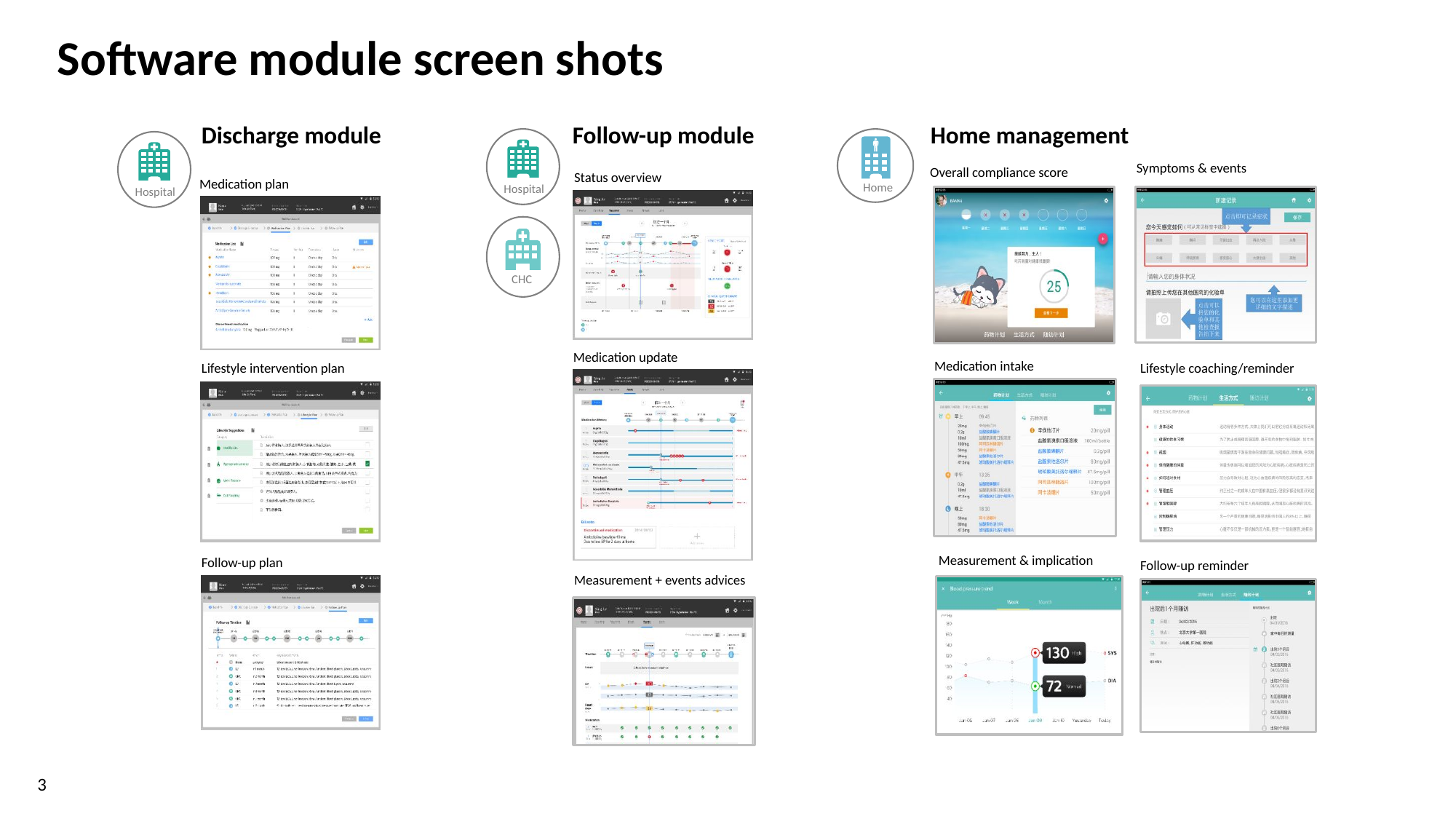

Software module screen shots
Discharge module
Follow-up module
Home management
Hospital
Home
Hospital
Symptoms & events
Overall compliance score
Status overview
Medication plan
CHC
Medication update
Medication intake
Lifestyle intervention plan
Lifestyle coaching/reminder
Measurement & implication
Follow-up plan
Follow-up reminder
Measurement + events advices
