# Supplementary material for: Effects on Adherence to a Mobile App–Based Self-management Digital Therapeutics Among Patients With Coronary Heart Disease: Pilot Randomized Controlled Trial
Source: JMIR Mhealth Uhealth. 2022 Feb 15;10(2):e32251. doi: 10.2196/32251 (PMC8889473; doi:10.2196/32251)
Supplement: Multimedia Appendix 3 [file mhealth_v10i2e32251_app3.pdf]

CONSORT-EHEALTH checklist (V.1.6.1): 2011 checklist of information to include when reporting ehealth/mhealth trials (web-based/Internet-based intervention and decision aids, but also social media, serious games, DVDs, mobile applications, certain telehealth applications) *

Section/Topic	Item No	CONSORT** Checklist item	Reported on page No
**Title and abstract**			
	1a	Identification as a randomised trial in the title	1
	1b	Structured summary of trial design, methods, results, and conclusions<br>NPT*** extension: Description of experimental treatment, comparator, care providers, centers, and blinding status	1
**Introduction**			
Background and objectives	2a	Scientific background and explanation of rationale	2
	2b	Specific objectives or hypotheses	2
**Methods**			
Trial design	3a	Description of trial design (such as parallel, factorial) including allocation ratio	2
	3b	Important changes to methods after trial commencement (such as eligibility criteria), with reasons	N/A
Participants	4a	Eligibility criteria for participants	2-3
	4b	Settings and locations where the data were collected	3
Interventions	5	The interventions for each group with sufficient details to allow replication, including how and when they were actually administered	3-4
Outcomes	6a	Completely defined pre-specified primary and secondary outcome measures, including how and when they were assessed	4
	6b	Any changes to trial outcomes after the trial commenced, with reasons	N/A
Sample size	7a	How sample size was determined<br>NPT: When applicable, details of whether and how the clustering by care provides or centers was addressed	4
	7b	When applicable, explanation of any interim analyses and stopping guidelines	4
Randomisation:			
Sequence generation	8a	Method used to generate the random allocation sequence<br>NPT: When applicable, how care providers were allocated to each trial group	3

	8b	Type of randomisation; details of any restriction (such as blocking and block size)	3
Allocation concealment mechanism	9	Mechanism used to implement the random allocation sequence (such as sequentially numbered containers), describing any steps taken to conceal the sequence until interventions were assigned	3
Implementation	10	Who generated the random allocation sequence, who enrolled participants, and who assigned participants to interventions	3
Blinding	11a	If done, who was blinded after assignment to interventions (for example, participants, care providers, those assessing outcomes) and how<br>NPT: Whether or not administering co-interventions were blinded to group assignment	N/A
	11b	If relevant, description of the similarity of interventions	4
Statistical methods	12a	Statistical methods used to compare groups for primary and secondary outcomes<br>NPT: When applicable, details of whether and how the clustering by care providers or centers was addressed	4
	12b	Methods for additional analyses, such as subgroup analyses and adjusted analyses	4
Ethics & Informed Consent	X26		2
**Results**			
Participant flow (a diagram is strongly recommended)	13a	For each group, the numbers of participants who were randomly assigned, received intended treatment, and were analysed for the primary outcome<br>NPT: The number of care providers or centers performing the intervention in each group and the number of patients treated by each care provider in each center	3-4
	13b	For each group, losses and exclusions after randomisation, together with reasons	3-4
Recruitment	14a	Dates defining the periods of recruitment and follow-up	3-4
	14b	Why the trial ended or was stopped [early]	N/A
Baseline data	15	A table showing baseline demographic and clinical characteristics for each group<br>NPT: When applicable, a description of care providers (case volume, qualification, expertise, etc.) and centers (volume) in each group	5
Numbers analysed	16	For each group, number of participants (denominator) included in each analysis and whether the analysis was by original assigned groups	3-5
Outcomes and estimation	17a	For each primary and secondary outcome, results for each group, and the estimated effect size and its precision (such as 95% confidence interval)	6
	17b	For binary outcomes, presentation of both absolute and relative effect sizes is recommended	6
Ancillary analyses	18	Results of any other analyses performed, including subgroup analyses and adjusted analyses, distinguishing	6-8

		pre-specified from exploratory	
Harms	19	All important harms or unintended effects in each group (for specific guidance see CONSORT for harms)	N/A
**Discussion**			
Limitations	20	Trial limitations, addressing sources of potential bias, imprecision, and, if relevant, multiplicity of analyses	8-9
Generalisability	21	Generalisability (external validity, applicability) of the trial findings<br>NPT: External validity of the trial findings according to the intervention, comparators, patients, and care providers or centers involved in the trial	8-9
Interpretation	22	Interpretation consistent with results, balancing benefits and harms, and considering other relevant evidence<br>NPT: In addition, take into account the choice of the comparator, lack of or partial blinding, and unequal expertise of care providers or centers in each group	8-9
**Other information**			
Registration	23	Registration number and name of trial registry	2
Protocol	24	Where the full trial protocol can be accessed, if available	2
Funding	25	Sources of funding and other support (such as supply of drugs), role of funders	N/A
Competing interests	X27		9

* Please view this document for an explanation of specific EHEALTH clarifications needed for each item.

**CONSORT = Consolidated Standards of Reporting Trials [10]

*** NPT = non pharmacological treatment (CONSORT extension) [11]

References

1. Baker TB, Gustafson DH, Shaw B, Hawkins R, Pingree S, Roberts L, Strecher V. Relevance of CONSORT reporting criteria for research on eHealth interventions. Patient Educ Couns. 2010 Dec;81 Suppl:S77-86
2. Talmon J, Ammenwerth E, Brender J, de Keizer N, Nykänen P, Rigby M. STARE-HI--Statement on reporting of evaluation studies in Health Informatics. Int J Med Inform. 2009 Jan;78(1):1-9. Epub 2008 Oct 18.
3. Eysenbach G. Issues in evaluating health websites in an Internet-based randomized controlled trial. J Med Internet Res 2002;4(3):e17
4. Blankers M, Koeter MWJ, Schippers GM. Missing Data Approaches in eHealth Research: Simulation Study and a Tutorial for Nonmathematically Inclined Researchers. J Med Internet Res 2010;12(5):e54
5. Eysenbach G. The law of attrition. J Med Internet Res 2005;7(1):e11
6. Proudfoot et al. Establishing Guidelines for Executing and Reporting Internet Intervention Research. Cognitive Behaviour Therapy (forthcoming)
7. Webb TL, Joseph J, Yardley L, Michie S. Using the Internet to Promote Health Behavior Change: A Systematic Review and Meta-analysis of the Impact of Theoretical Basis, Use of Behavior Change Techniques, and Mode of Delivery on Efficacy. J Med Internet Res 2010;12(1):e4
8. Cugelman B, Thelwall M, Dawes P. Online Interventions for Social Marketing Health Behavior Change Campaigns: A Meta-Analysis of Psychological Architectures and Adherence Factors. J Med Internet Res 2011;13(1):e17
9. Eysenbach G. Improving the Quality of Web Surveys: The Checklist for Reporting Results of Internet E-Surveys (CHERRIES). J Med Internet Res 2004;6(3):e34
10. Schulz KF, Altman DG, Moher D, for the CONSORT Group (2010) CONSORT 2010 Statement: Updated Guidelines for Reporting Parallel Group Randomised Trials. PLoS Med 7(3): e1000251
11. Boutron I, Moher D, Altman DG, Schulz K, Ravaud P, for the CONSORT group. Extending the CONSORT Statement to randomized trials of nonpharmacologic treatment: explanation and elaboration. Ann Intern Med. 2008:295-309
